# Genomic insights into the mitochondria of 11 eastern North American species of *Cladonia*

**DOI:** 10.1080/23802359.2018.1463827

**Published:** 2018-04-26

**Authors:** Laurel M. Brigham, Luis M. Allende, Benjamin R. Shipley, Kayla C. Boyd, Tanya J. Higgins, Nicholas Kelly, Carly R. Anderson Stewart, Kyle G. Keepers, Cloe S. Pogoda, James C. Lendemer, Erin A. Tripp, Nolan C. Kane

**Affiliations:** a Department of Ecology and Evolutionary Biology, University of Colorado, Boulder, CO, USA;; b Department of Molecular, Cellular and Developmental Biology, University of Colorado, Boulder, CO, USA;; c Institute of Systematic Botany, The New York Botanical Garden, New York, NY, USA;; d Museum of Natural History, University of Colorado, Boulder, CO, USA

**Keywords:** *Cladonia*, genome, lichen, mitochondrion, symbiosis

## Abstract

*Cladonia* is among the most species-rich genera of lichens globally. Species in this lineage, commonly referred to as reindeer lichens, are ecologically important in numerous regions worldwide. In some locations, species of *Cladonia* can comprise the dominant groundcover, and are a major food source for caribou and other mammals. Additionally, many species are known to produce substances with antimicrobial properties or other characteristics with potentially important medical applications. This exceptional morphological and ecological variation contrasts sharply with the limited molecular divergence often observed among species. As a new resource to facilitate ongoing and future studies of these important species, we analyse here the sequences of 11 *Cladonia* mitochondrial genomes, including new mitochondrial genome assemblies and annotations representing nine species: *C. apodocarpa, C. caroliniana, C. furcata, C. leporina, C. petrophila, C. peziziformis, C. robbinsii, C. stipitata,* and *C. subtenuis*. These 11 genomes varied in size, intron content, and complement of tRNAs. Genes annotated within these mitochondrial genomes include 15 protein-coding genes, the large and small ribosomal subunits (mtLSU and mtSSU), and 23–26 tRNAs. All *Cladonia* mitochondrial genomes contained *atp9,* an important energy transport gene that has been lost evolutionarily in some lichen mycobiont mitochondria. Using a concatenated alignment of five mitochondrial genes (*nad2*, *nad4*, *cox1*, *cox2*, and *cox3*), a Bayesian phylogeny of relationships among species was inferred and was consistent with previously published phylogenetic relationships, highlighting the utility of these regions in reconstructing phylogenetic history.

## Introduction

*Cladonia* is a charismatic genus of lichen-forming ascomycetes in the order Lecanorales (Miadlikowska et al. 2014). Globally, the genus contains upwards of 300 species (Ahti 1998; Stenroos and DePriest 1998; Stenroos et al. 2002; Kirk 2017), most of which are characterized by dimorphic thalli that consist of a squamulose primary thallus and a fruticose secondary thallus (Ahti 1998). In some groups, such as the true reindeer lichens that were historically classified in *Cladina*, the primary thallus is ephemeral and crustose (Thomson 1967; Ahti 1998). In other groups, including a clade of three eastern North American endemic species (*C. apodocarpa, C. petrophila*, and *C. stipitata*), the fruticose secondary thallus is reduced to a short stipe or is entirely lacking (Lendemer and Hodkinson 2009).

This morphological variation is mirrored in the chemical diversity within the genus, which spans a wide variety of secondary metabolites (Rundel 1969; Ahti 1998; Nash et al. 2002), many among which have been inferred to have pharmacological importance (Lauterwein et al. 1995; Zhang et al. 2012; Kosanić et al. 2014; Ramos et al. 2014). The visually striking thalli of *Cladonia* frequently comprise the dominant groundcover in diverse habitats including deserts, temperate forest glades, and boreal forests. In the latter, *Cladonia* are the major food source for ungulates such as reindeer, solidifying their role as a vital component of ecosystem function (Ahti 1998; Stenroos and DePriest 1998; Stenroos et al. 2002; Kirk 2017). Members of the genus grow in a range of moisture and light conditions, reflecting the broad ecological amplitude of the genus as a whole (Cringan 1957; Ahti and Hepburn 1967; Corns 1983; Meades and Moores 1989).

Despite widespread occurrence and exceptional morphological and ecological variation among species of *Cladonia* (Osyczka et al. 2014; Pino-Bodas et al. 2015), relatively few genetic resources have been developed for the genus (e.g. *Cladonia grayi*; Joneson et al. 2011). Here, we present annotated mitochondrial genomes of nine species of *Cladonia*: *C. apodocarpa, C. caroliniana, C. furcata, C. leporina*, *C. petrophila, C. peziziformis*, *C. robbinsii*, *C. stipitata,* and *C. subtenuis*. Of these, six (*C. apodocarpa, C. caroliniana, C. leporina, C. petrophila, C. robbinsii,* and *C. stipitata*) are endemic to eastern North America where they (and the remaining three species) are especially abundant in the southern Appalachian Mountains (Fink 1935; Thomson 1967; Hale 1969; Ahti 1984; Brodo et al. 2001; Lendemer and Hodkinson 2009; Lewis 2014; Tripp and Lendemer 2019 [in press]; Tripp et al., in review). To enable future comparative evolutionary and genomic analyses within genus *Cladonia,* we analysed differences in genome size and protein coding gene content, characterized intron-encoded retrotransposable elements, and conducted Bayesian analyses of five mitochondrial genes to assess efficacy of using these loci in phylogenetic reconstruction.

## Methods

Samples of *Cladonia apodocarpa* (voucher: *Lendemer 48789*; Lat/long 35.0253, –83.1747; NCBI Accession MG958507)*, C. caroliniana* (voucher: *Lendemer 49894*; Lat/long 34.3291, –87.5092; NCBI Accession MG708277)*, C. furcata* (voucher: *Tripp 6423 & Lendemer*; Lat/long 34.9555, –85.9996; NCBI Accession MG711314)*, C. leporina* (voucher: *Tripp 6543 & Lendemer*; Lat/long 34.3291, –87.5092; NCBI Accession MG725377), *C. petrophila* (voucher: *Lendemer 49138 & Tripp*; Lat/long 34.4717, –86.0511; NCBI Accession MG941021)*, C. peziziformis* (voucher: *Tripp 6281 & Lendemer*; Lat/long 34.4559, –85.5845; NCBI Accession MG686615), *C. robbinsii* (voucher: *Lendemer 49897 & Tripp*; Lat/long 34.3291, –87.5092; NCBI Accession MG725618), *C. stipitata* (voucher: *Tripp 6060 & Lendemer*; Lat/long 35.4835, –83.8148; NCBI Accession MG851822), and *C. subtenuis* (voucher: *Lendemer 49895 & Tripp*; Lat/long 34.3291, –87.5092; NCBI Accession MG949117) were collected by J.C. Lendemer, E.A. Tripp, K.G. Keepers, K.H. White, C.R. Anderson Stewart, J.R. Hoffman, and C.M. McCain from the Southern Appalachian Mountains of North Carolina and Alabama, U.S.A. between 2015 and 2017. Museum voucher specimens are housed at the New York Botanical Garden (NY Herbarium) and University of Colorado (COLO Herbarium).

Genomic DNA was extracted using a Qiagen DNeasy 96 plant kit, with the protocol modified to include a 10 minute 65 °C incubation step for the ground material in lysis buffer and a 100% ethanol wash before final drying of the membrane prior to elution. Genomic libraries were prepared using Nextera^®^ XT DNA library prep kits (Illumina^®^) and each sample was barcoded by the unique dual index adapters Nextera^®^ i5 and i7. Samples that passed QC were processed for paired-end 150 base pair reads on the Illumina NextSeq^®^ sequencer at the University of Colorado’s BioFrontiers Institute Next-Generation Sequencing Facility in Boulder, Colorado. Genome assemblies and annotations were conducted by undergraduate and graduate students enrolled in N. Kane’s Genomics class at the University of Colorado, Boulder, with additional assistance and careful verification performed by more experienced graduate students and faculty.

Genomic reads were trimmed using Trimmomatic-0.36 with the parameters: ‘ILLUMINACLIP:NexteraPE-PE.fa:2:20:10 MINLEN:140 LEADING:20 TRAILING:20’ (Bolger, et al 2014). Reads were assembled *de novo* using SPAdes v. 3.9 (Bankevich et al. 2012), annotated using DOGMA (Wyman et al. 2004) and Chlorobox (Tillich et al. 2017), and finished in Sequin v. 15.10 from NCBI (Bethesda, MD) for final submission to GenBank.

### Phylogenetic analysis

The phylogeny of nine species of *Cladonia* was inferred using sequences from the mitochondrial genes *cox3*, *nad2*, *nad4*, *cox1*, and *cox2* annotated in this study as per Funk et al. (2018). To test monophyly of these nine species of *Cladonia* against outgroups and to further place this lineage in the context of relatives, we downloaded sequences representing these genes from other taxa from GenBank, all of which derived from mitochondrial genomes that we and student colleagues assembled in earlier work, as follows: *Alectoria fallacina* [MG711470], *Arthonia susa* [MH015348], *Cladonia rangiferina* [KY460674], *Cladonia uncialis* [KY352404], *Coccocarpia palmicola* [NC_034332], *Gomphilus americanus* [NC_034790], *Graphis lineola* [KY315996], *Heterodermia speciosa* [KY328643], *Hypogymnia vittata* [KY362374], *Icmadophila ericetorum* [KY124637], *Imshaugia aleurites* [KY352227], *Lepraria oxybapha* [KY348846], *Leptogium hirsutum* [NC_034928], *Menegazzia subsimilis* [KY352491], *Mycocalicium subtile* [KY348761], *Opegrapha vulgata* [KY315997], *Parmotrema stuppeum* [KY362439], *Pertusaria ostiolata* [KY346830], *Phlyctis boliviensis* [KY305663], *Pertusaria corallina* [NC_034779], *Usnea pensylvanica* [KY321923], *U. ceratina* [KX987159], and *U. cornuta* [KY100278]. The same five genes (*cox1*, *cox2*, *cox3*, *nad2*, and *nad4*) were extracted from the above accessions. Bayesian phylogenetic analyses were conducted on the individual trees and there was no conflict between any resultant trees (Mason-Gamer and Kellogg 1996). To improve support in the final tree, we concatenated the five genes using Muscle aligner (Edgar 2004) and then manually curated the alignment. Gaps were treated as missing data and regions in which more than one taxon was missing data were excluded from the alignment (available on Zenodo 1206280). The optimal model of GTR + Γ+I was chosen using an Akaike information criterion (AIC) selected in ModelFinder (Kalyaanamoorthy et al. 2017), and applied to phylogenetic analysis. Bayesian topologies were inferred in MrBayes (Huelsenbeck and Ronquist 2001; Ronquist and Huelsenbeck 2003), sampling trees over 75,000 MCMC generations (Nei and Kumar 2000) excluding 25,000 burn-in generations, with separate partitions specified for each of the five genes. Bayesian posterior probabilities were mapped onto a resultant 50% majority rule consensus Bayesian tree (Tamura and Nei 1993). This tree was rooted using *M. subtile* [Class Eurotiomycetidae] as well as *A. susa* and *O. vulgata* [Class Arthoniomycetidae] as outgroups.

## Results and discussion

### Mitochondrial genome content and organization

The nine new genomes presented here broaden our understanding of mitogenome structure within *Cladonia* given that prior to this study there existed only two mitochondrial genomes from the genus (*C. rangiferina, C. uncialis*) available on GenBank. The size of these nine genomes varied from 45,312 bp (*C. peziziformis*) to 60,062 bp (*C. stipitata*) with a mean of 53,407 bp and standard deviation of 5307.8 bp. A large inversion block involving the genes *nad6*, *cox3*, *LSU*, *nad2*, and *nad3* was previously documented in *C. uncialis* (Pogoda et al. 2018), however, this inversion was not located in any of the newly analysed genomes. Similarly, no other genomic rearrangements were detected in the nine genomes, demonstrating a conserved synteny in these representatives of *Cladonia*. Each mitogenome contained between 23 and 26 tRNAs and their locations were consistent among the species analysed. An exception was *trnK*, wherein an additional copy of *trnK* was observed in *C. apodocarpa, C. petrophila*, and *C. stipitata*, located between the *cox1* and *nad1* genes. All nine mitogenomes contained 15 protein-coding genes (*cob*, *cox1*, *cox2*, *cox3*, *nad1*, *nad2*, *nad3*, *nad4*, *nad4L*, *nad5*, *nad6*, *atp6*, *atp8*, *atp9*, and *rps3*). Notably, *atp9*, a key mitochondrial gene involved in energy production that has been lost in some lichen lineages, including members of the family Parmeliaceae (Pogoda et al. 2018), was detected in all nine *Cladonia*. The presence of *atp9* in our newly analysed mitogenomes is consistent with earlier results that detected this gene in two species of *Cladonia* (Pogoda et al. 2018).

### Introns and intergenic spacer regions

Variation in intron position and size, particularly in *cox1*, accounted for the majority of variation in mitochondrial genome size. In all species except *C. peziziformis* that encoded *cox1* as a single exon, introns in *cox1* contained LAGLIDADG and GIY-YIG homing endonucleases. Presence of these selfish, parasitic elements in lichen mitochondrial genomes has been previously documented (Pogoda et al. 2018) and they are thought to aid in the mobility of their host introns (Guha et al. 2017). All species examined contained introns in *mtLSU*, and *C. stipitata* and *C. leporina* also contained introns in the *mtSSU*.

### Phylogenetic history

**Figure 1.**
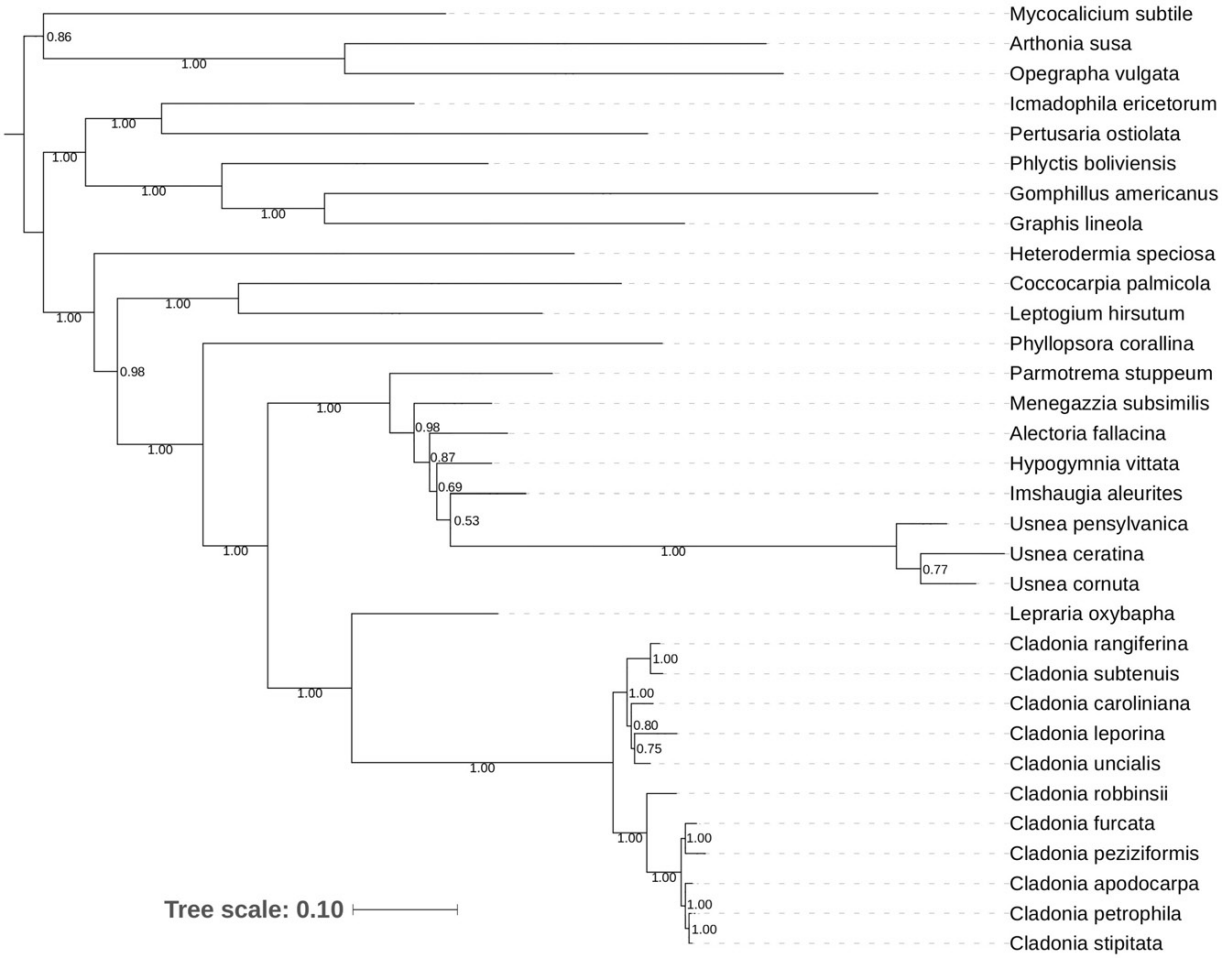
Majority rule consensus tree inferred from Bayesian analyses of five concatenated mitochondrial genes (*cox3*, *nad2*, *nad4*, *cox1*, *cox2*) from 11 *Cladonia* species (data herein assembled) plus several additional taxa whose sequences were downloaded from GenBank (and originally generated by the authors). Numbers indicate posterior probabilities. Branch lengths represent the number of substitutions per site per unit time (scale below). Tree was rooted using *M. subtile* [Eurotiomycetidae] as well as *A. susa* and *O. vulgata* [Arthoniomycetidae].

Our molecular phylogenetic analyses (Figure 1) resolved all *Cladonia* species examined in this study as monophyletic (PP = 1.00) and furthermore, partitioned into two clades. In particular, taxa formerly ascribed to the genus *Cladina* together with species of *Cladonia* sect. *Unciales* (i.e. *C. subtenuis, C. rangiferina, C. caroliniana, C. leporina, C. uncialis*) were inferred to form a strongly supported clade (‘Clade 1’ herein; (PP = 1.00)) whereas species in *Cladonia* sensu stricto (i.e. species that are predominantly squamulose or podetiate including *C. robbinsii, C. peziziformis, C. apodocarpa, C. petrophila, C. stipitata, C. furcata*) formed a second strongly supported clade (‘Clade 2’ herein; (PP = 1.0)). Overall, relationships were concordant with trees produced using ribosomal DNA as well as chemical and morphological data (Stenroos et al. 2002), which included 10 of the 11 *Cladonia* represented here (*C. stipitata* was not included in that study).

## Conclusions

The newly sequenced, assembled, and annotated *Cladonia* mitogenomes exhibited a moderate range in size, with intronic and intergenic regions of variable lengths associated with changes in genome size. These mitochondrial genomes all contained the same set of 15 protein-coding genes. Results from our study confirm earlier findings (Pogoda et al. 2018) that species of *Cladonia* have retained a key gene involved in energy production, *atp9*. Together, these data provide important genetic resources for these charismatic, morphologically variable and ecologically important lichens, as well as new resources for further study of lichen mitochondrial genome evolution and co-evolution with their obligate symbiotic partners (Tripp et al. 2017).
